# ThrombUS+ Project: Toward Wearable Continuous Point-of-Care Monitoring for Deep Vein Thrombosis of the Lower Limb

**DOI:** 10.34133/csbj.0082

**Published:** 2026-05-27

**Authors:** Vaidotas Marozas, Stylianos Didaskalou, Rimvydas Eitminavičius, Nick Portokallidis, Rytis Jurkonis, Saulius Daukantas, Mantas Jucevičius, Andrius Rapalis, Nicolas Pousset, Mathieu Legros, Sean Toffessi Siewe, Nicolas Lange, Sören Köble, Marco Kircher, Andrius Sakalauskas, Pavlos Moustakidis, Susann Balling, Jürgen Querengässer, Lara Alessia Moltani, Jessica Cesana, Sami Maja, Roni Ahola, Andrius Macas, Neringa Balčiūnienė, Rytis Kijauskas, Adomas Aladaitis, Linas Velička, Michail Potoupnis, Elvira Grandone, Maxime Gautier, Thorsten Prinz, Sophia Zoe Narten, Pietro Dionisio, Spiros Anagnostopoulos, Lucas Javier Segal, Federico Puppo, Katerina Pavlidi, Arūnas Lukoševičius, Antti Vehkaoja, Eleni Kaldoudi

**Affiliations:** ^1^ Kaunas University of Technology, Kaunas 44029, Lithuania.; ^2^ ATHENA RC, Xanthi 67100, Greece.; ^3^ Vermon Sa, Tours 37000, France.; ^4^ Fraunhofer Institute for Photonic Microsystems IPMS, Dresden 01109, Germany.; ^5^ Telemed UAB, Vilnius 02301, Lithuania.; ^6^ Echonous Inc., Redmond, WA 98052, USA.; ^7^ Medis Medizinische Messtechnik Gmbh, Ilmenau 98693, Germany.; ^8^ Comftech Srl, Monza 20900, Italy.; ^9^ Tampere University, Tampere 33100, Finland.; ^10^ Lithuanian University of Health Sciences, Kaunas 44307, Lithuania.; ^11^ General Hospital Papageorgiou, Thessaloniki 54603, Greece.; ^12^ Fondazione Casa Sollievo Della Sofferenza, San Giovanni Rotondo FG 71013, Italy.; ^13^ Groupement Hospitalier Eaubonne Montmorency Simone Veil, Montmorency 95160, France.; ^14^ VDE Association for Electrical, Electronic and Information Technologies, Offenbach Am Main 63069, Germany.; ^15^ Medea Srl, Massa (MS) 54100, Italy.; ^16^ Phaze SA, Athens 15451, Greece.; ^17^ PredictBy Research and Consulting S.L, Barcelona 08018, Spain.; ^18^ SciGen SA, Xanthi 67100, Greece.

## Abstract

Deep vein thrombosis (DVT) is characterized by the formation of blood clots in the deep veins, most commonly in the lower limbs, obstructing blood flow. In about 50% of cases, the clot dislodges and travels to the lungs, causing potentially fatal pulmonary embolism. Diagnosing DVT is challenging, as up to half of cases are asymptomatic. Early and accurate diagnosis is critical, but the current state of the art in ultrasound and plethysmography is insufficient for continuous, operator-independent, point-of-care monitoring. This paper introduces the concept and advances of the ThrombUS+ Horizon Europe project, which aims to innovate toward modular and wearable system for continuous, point-of-care DVT monitoring. The system leverages a multimodal approach, incorporating compression ultrasonography, electrical impedance plethysmography, and light reflection rheography, by re-engineering these clinical technologies for seamless integration into a wearable architecture. Manual compression of an ultrasound transducer is replaced by a sophisticated actuator, enabling precise pressure control via a piezoelectric micropump with feedback signals. The same actuator supports plethysmography modules, providing additional data for DVT assessment. These inputs, combined with physical movement monitoring, are processed by an artificial intelligence-driven central intelligence unit for DVT detection, risk assessment, and prevention. Preliminary laboratory testing of the individual modules highlights the system’s potential for autonomous DVT monitoring and detection. The ThrombUS+ uses big datasets for machine learning training collected in the project via 3 large-scale clinical studies. The entire system will be validated in the clinical setting via an early feasibility study and a multicenter clinical trial.

## Graphical Abstract

**Figure:**
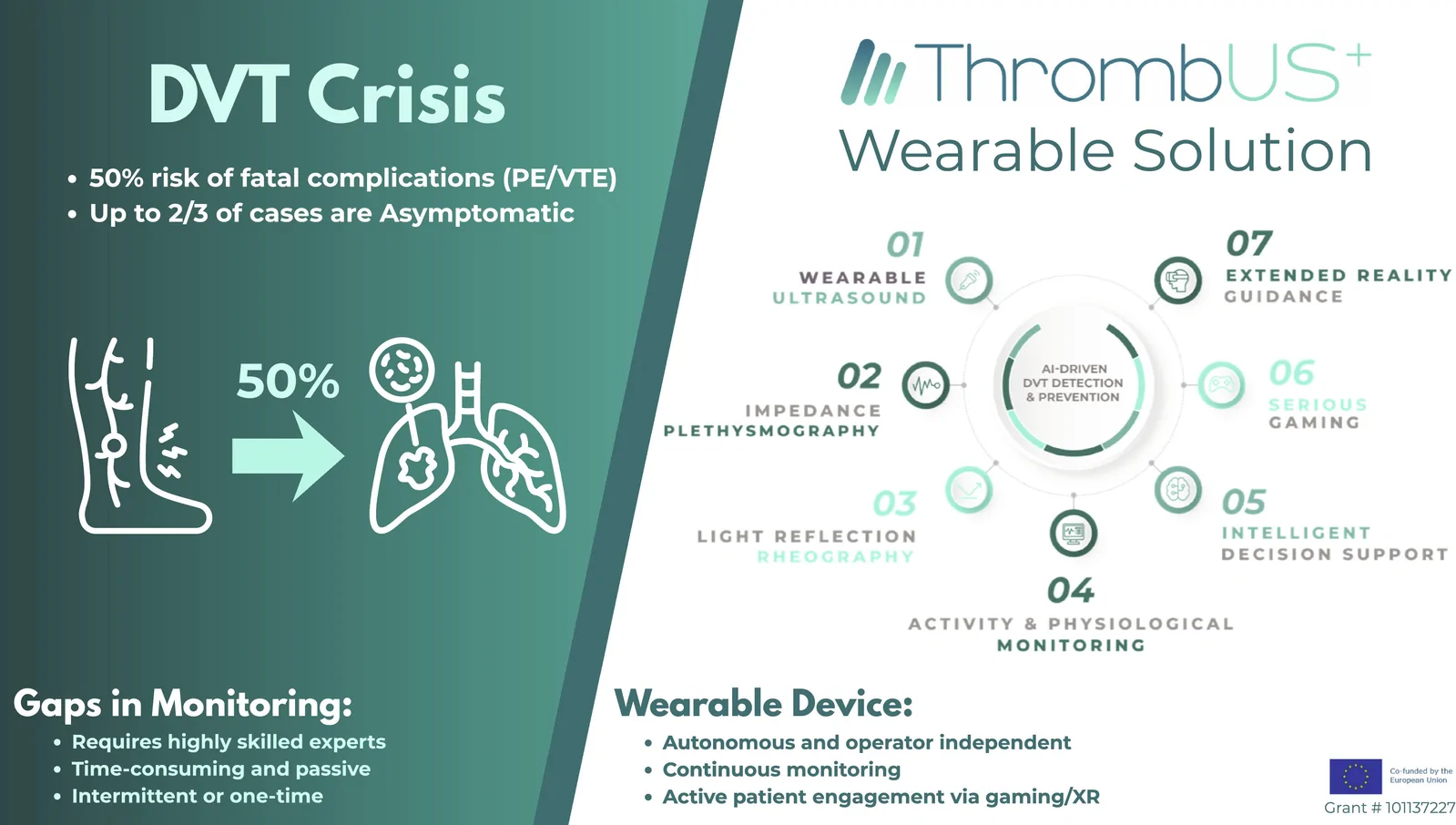

## Introduction

Deep vein thrombosis (DVT) is the condition of blood clot formation within one or more deep veins of the body. Blood clots are mostly composed of fibrin, red blood cells, platelets, leukocytes, and other cellular compounds [1] and can form in a matter of seconds to minutes [2]. Most of the blood clots form in the deep veins of the lower limbs, but the likelihood of involvement at a specific site by anatomical location differs: distal veins 40%, popliteal 16%, femoral 20%, common femoral 20%, and iliac veins 4% [3]. Clots can detach from their primary formation site and block an artery in the lungs, causing a life-threatening condition known as pulmonary embolism (PE). Nearly half of patients with proximal DVT develop PE, and approximately one-quarter of patients with PE may not survive, sometimes without preceding clinical symptoms [4,5]. Notably, proximal DVT (above the knee) poses a higher risk of embolization [6,7]. Another long-term complication of DVT is the post-thrombotic syndrome that affects almost 20% to 50% of patients. Post-thrombotic syndrome symptoms include pain, edema, and skin discoloration that potentially may lead to venous ulcers [8]. Therefore, early diagnosis and treatment of DVT are crucial to prevent potentially fatal complications or deterioration of quality of life.

It is estimated that venous thromboembolism disorders represent the third most common cause of vascular mortality in the world after heart attacks and stroke [9]. The annual incidence rates for PE and DVT range from 39 to 115 and from 53 to 162 per 100,000, respectively [10]. Moreover, studies emphasize that incidence and prevalence are often underestimated, as DVT and resulting pulmonary emboli frequently go undiagnosed because of the asymptomatic nature of this condition [9]. PE causes about 300,000 deaths in the United States annually [10]. In contrast, deaths related to venous thromboembolism are estimated to be around 370,000 in 6 European countries in 2004, with a third of them dying suddenly, before therapy can be initiated [10].

The pathophysiology of DVT primarily involves 3 factors that contribute primarily to clot formation: endothelial injury, venous stasis, and blood hypercoagulability [9]. Long periods of immobility, such as after long hospitalization or even long airplane flights, reduce blood flow in the veins of the lower limbs, which can promote blood clot formation. Surgery, trauma, or inflammation can all cause damage to vessels and trigger blood coagulation and clot formation. Finally, certain conditions, such as cancer, thrombophilia, or pregnancy, as well as the use of contraceptives, may increase the risk of DVT.

Prophylaxis against DVT has been emphasized in the rehabilitation setting to prevent its fatal complications. There are several DVT prophylaxis methods: anticoagulants, graduated compression stockings, intermittent pneumatic compression, neuromuscular electrical stimulation, active leg exercises, and exoskeletons for performing passive leg exercises. Exergaming was also shown to be a promising method for DVT prevention by motivating patients to engage in rehabilitation exercises [11].

Although contrast venography remains the historical reference standard, compression ultrasound (CUS) is now universally recognized as the clinical gold standard for DVT diagnosis due to its noninvasive nature and high diagnostic accuracy [12–14]. During this imaging test, medical experts assess vein compressibility by applying gentle pressure with the ultrasound probe. If the vein lumen does not fully collapse, it indicates the presence of a clot. Compression ultrasonography is performed along the deep veins of the entire lower limb with compressions every 1 to 2 cm. Alternatively, CUS can be performed in only 2 major anatomical sites, such as the common femoral vein at the groin and the popliteal vein, while other approaches include a third anatomical site, i.e., the femoral vein. Those 2- or 3-point methods are simple and quick; however, a repeat test is mandatory within a week to exclude unidentified potential distal DVT [15].

Currently, most ultrasound scans are performed in the radiology or cardiovascular departments by highly trained medical experts. Artificial intelligence (AI) emerges as a powerful tool to assist the nonexpert ultrasound operator by providing real-time guidance during imaging acquisition and assisting in interpreting the CUS examination. The integration of AI into portable ultrasound scanners enables nonexperts to perform point-of-care assessments, facilitating the detection of DVT outside traditional settings [16,17]. Early detection, however, requires continuous vigilance and frequent examination.

Alternative, noninvasive, and easily deployable methods to assess venous capacity and flow indicating blockage that may be due to DVT include venous occlusion plethysmography (VOP) [18] and light reflection rheography (LRR) [19,20]. A meta-analysis noted that although plethysmography and rheography methods offer additional diagnostic value, they are not suitable as a stand-alone test because they lack standardization across testing methodologies and reported outcomes [21].

The ThrombUS+ project aims to develop a wearable device for autonomous, point-of-care, and continuous patient monitoring, revolutionizing the early detection and management of DVT. The system features a novel wearable ultrasound probe, capable of performing AI-driven operator-independent CUS. Additionally, a network of wearable sensors will enable intermittent, operator-free DVT monitoring using electrical impedance plethysmography and LRR. Finally, integrated inertial measurement units in combination with an extended reality (XR) and serious gaming (SG) platform provide real-time guidance and motivation for patients to perform various lower limb exercises for DVT prevention. Emphasis is also placed on sustainability through the design of a low-cost, accessible device that considers the entire system life cycle. Finally, the project aims to promote open science by reducing redundancy, disseminating knowledge, and fostering transparency and accountability. The concept of the ThrombUS+ system is presented in Fig. 1.

**Fig. 1.**
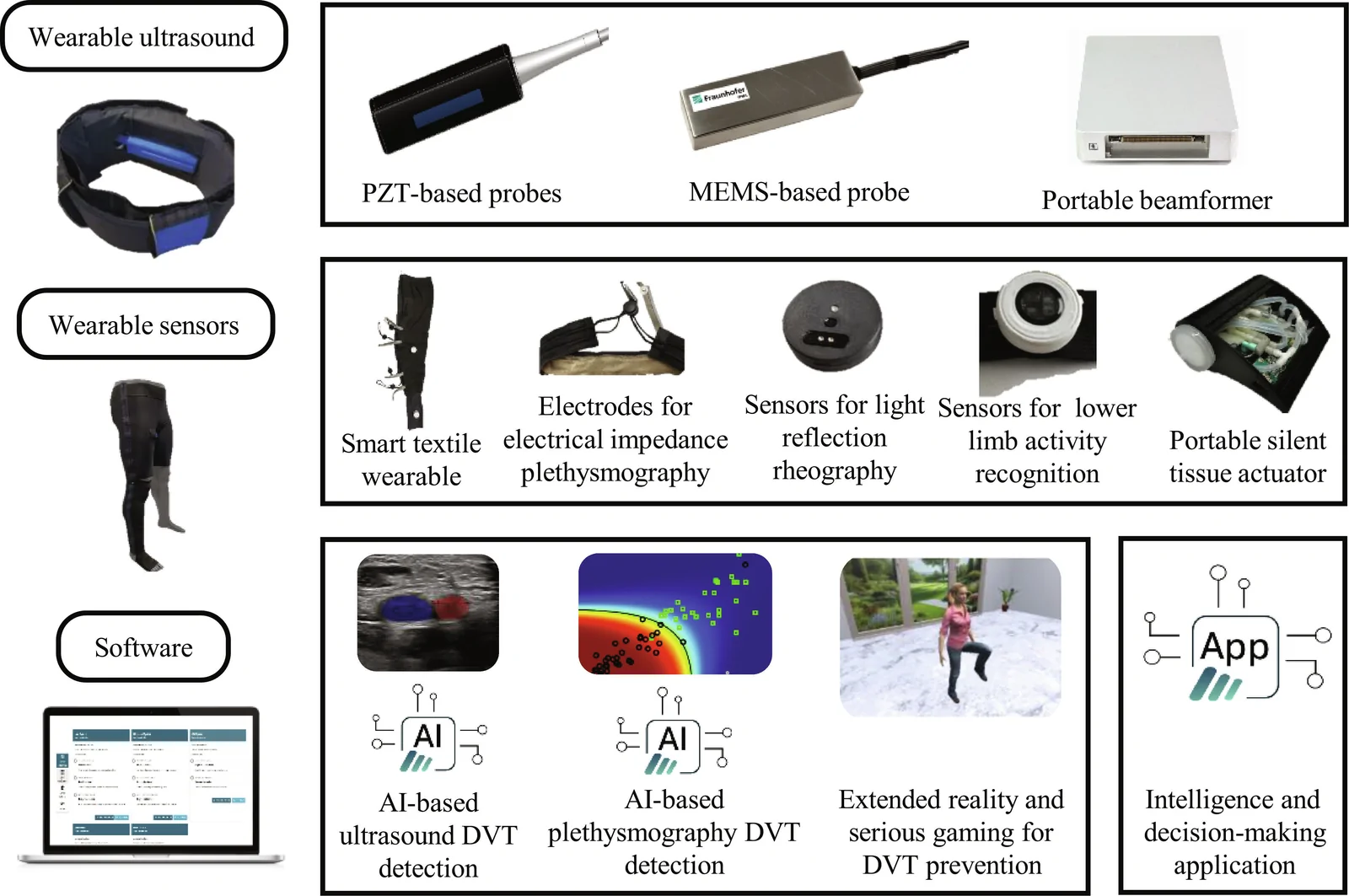
Overview of the proposed wearable for continuous monitoring of the lower limb for early DVT detection. The device comprises a wearable strap, a network of sensors and software. The strap integrates a wearable ultrasound device with air bladders for implementing the compression ultrasound (CUS) method. Electrical impedance, light reflection, and activity sensors, along with a tissue actuator, will be integrated into smart textiles, creating a wearable network of sensors. An intelligence and decision-making application coordinates the continuous monitoring, assessment, and prevention of deep vein thrombosis (DVT), utilizing artificial intelligence (AI) models, an extended-reality environment, and serious gaming.

## Innovation beyond the State of the Art

The ThrombUS+ project advances DVT management by shifting from operator-dependent diagnostics in the radiology department to autonomous and continuous monitoring at the point of care, introducing several key innovations:

**Continuous monitoring modality for DVT detection.** The primary goal of pharmacologic anticoagulation prophylaxis is to prevent fatal PE. In the intensive care unit and surgical patients, pharmacologic prophylaxis is associated with a reduction in mortality [22,23]. However, all anticoagulant strategies for venous thromboprophylaxis increase the risk of bleeding. In high-risk patients, such as those undergoing major (i.e., orthopedic) surgery or who are immobilized, the risk of DVT remains clinically important despite prophylaxis and preventive measures [24]. ThrombUS+ proposes advancement of proven DVT detection modalities, namely, ultrasound imaging and plethysmography measurements, beyond their current state of the art. The project aims to create an integrated, operator-free device for compression and Doppler ultrasound combined with plethysmography measurements, for continuous DVT monitoring. This allows for the ongoing monitoring of high-risk patients, including those who are postoperative, during long surgeries, bedridden, or in pregnancy and postpartum stages.

**Operator-free DVT detection at the point of care.** Ultrasound exams (in DVT and in general) are performed by an ultrasonography technologist and read by a radiologist. Ultrasound is a proven, recommended modality for bedside point-of-care imaging in a multitude of situations [25]. Although ultrasound technology is comparatively cheap and commonly available, the dependence on expert operators often hinders the deployment of the modality in point-of-care settings. Thus, current research worldwide strives to exploit AI to create ultrasound probes that can be driven via a mobile phone and used without an expert operator to perform a diagnosis. In particular, a recent systematic review identified several research attempts employing deep learning approaches to improve diagnostic accuracy, automate image analysis, and support clinical decision-making in thrombosis management [26]. ThrombUS+ takes this approach a step further by proposing a wearable, operator-free, ultrasound system to perform CUS and DVT detection.

**Innovation in ultrasound transducer arrays for wearable, compact size, low-consumption imaging probes**. The emergent need for continuous, operator-free ultrasound imaging has pushed recent research and innovation toward wearable ultrasound probes. A notable example is the first announcements of experimental ultrasound patches for continuous heart monitoring [27,28]. ThrombUS+ major innovation is positioned exactly at the verge of a paradigm shift in ultrasound imaging toward continuous, wearable monitoring. The ThrombUS+ project plans a wearable ultrasound probe for continuous lower limb vascular imaging, including innovation on the tissue compression actuator required to realize compression imaging for DVT detection.

**Wearable, operator-free plethysmography.** The high prevalence and serious consequences of DVT have led to the development of several noninvasive diagnostic tools based on plethysmography [29]. This family of techniques measures changes in the limb size caused by tissue fluid or pooled blood in the veins. Electrical impedance plethysmography (EIP) applies a weak high-frequency electrical current via circumferential electrodes and measures impedance changes that reflect venous refill. LRR sheds an infrared light and measures the amount reflected by peripheral blood vessels, giving a model-based estimate of venous refill in deep veins. Although plethysmography performs well in comparison to ultrasound [30], it requires careful positioning of the patient’s leg and a compression cuff with rapid inflation and deflation times, and trained personnel to deduce DVT diagnosis from refill curves, which makes the method operator-dependent and not particularly easy to use. The ThrombUS+ project will use smart textile technology to embed electrodes and light sources/sensors in a wearable to create an operator-free, wearable system for plethysmography, DVT biomarker measurement, and DVT detection powered by AI.

**Silent cuff for comfortable and precise tissue compression.** Both conventional DVT ultrasound and plethysmography methods require tissue compression. In ultrasound, this is performed by the operator’s hand, while in plethysmography, by a compression cuff with rapid inflation/deflation rates. The ThrombUS+ project will use state-of-the-art, off-the-shelf piezoelectric micropump technology [31] to develop a compact, low consumption, and silent tissue compression actuator with characteristics that meet the requirements for precise pressure micro-control for ultrasound imaging and rapid inflation/deflation for plethysmography.

**Trustworthy and explainable AI for medical imaging diagnosis ported to support automated DVT detection.** Medical image analysis and automated diagnosis are among the leading applications of AI in healthcare [32]. Several of these AI algorithms are now approved by the Food and Drug Administration, with a few even coupled to reimbursements, such as the US Centers for Medicare and Medicaid Services payment for AI-enabled stroke detection and the New Technology Add-on Payments for AI services. Lightweight AI is the method of choice for operator-free, point-of-care automated diagnosis based on mobile devices [26]. ThrombUS+ will develop deep learning models based on big image/signal datasets obtained during the project to achieve automated biomarker measurements and automated DVT detection.

**Smart textiles and active garments for continuous DVT monitoring, risk estimation, and prevention.** Smart electronic textiles [33] and flexible sensors are currently in the spotlight with continuous advancements that recently provoked the outlining of a comprehensive roadmap [34]. ThrombUS+ capitalizes on patented smart textile technology to create wearable, textile-embedded sensors and compression actuators for DVT ultrasound detection and plethysmography measurements. Currently available physiological measurement and activity sensor networks will also be exploited in the project for patient status detection and XR physical exercise guidance for DVT prevention and rehabilitation.

**XR and SG for DVT prevention and rehabilitation.** XR applications in combination with SG offer promising solutions to improve the quality of life for bedridden patients in the clinical ward, making rehabilitation more engaging and interactive by encouraging users to participate in physical activities [35]. DVT prevention requires designing personalized activity plans with ongoing effort over an extended period [11]. In this regard, one of the biggest challenges is to accurately track patient activity patterns and assess their compliance with activity plans. This involves integrating sensors and other monitoring devices to collect data on patient movements, which can be challenging to implement and calibrate in the clinical environment [36]. ThrombUS+ leverages the wearable lower limb activity monitoring system and motion recognition models to implement an XR and SG platform to ensure technical accuracy and enhance adherence to prescribed physiotherapy protocols, facilitating effective rehabilitation independent of direct clinical supervision.

## Overall Methodology

The project relies on research and innovation in different areas to realize the novel components comprising the ThrombUS+ wearable and the driving decision-making module. The design is driven by a human-centered approach, while all stages of research and implementation follow a compliance-by-design approach, with regulatory requirements being built into the system development.

### Ultrasound system for wearable, automated CUS

The development of wearable probes is constrained by strict requirements for miniaturization and a low profile, as well as by requirements of medical vascular imaging, i.e., central frequency within the range of 5 to 7 MHz, a 4-MHz bandwidth, and a maximum penetration of 8 cm. Two piezoelectric architectures are investigated: a linear array for single-plane imaging and a novel row–column array offering simultaneous bi-plane imaging. In parallel, capacitive micromachined ultrasonic transducers (CMUTs) are investigated as a micro-electromechanical systems (MEMS)-based alternative. CMUT technology leverages wafer-scale fabrication to achieve high-volume, low-cost production and high energy efficiency.

The electronics layer involves the design of portable beamformer printed circuit boards. New real-time beamforming and multiplanar scanning algorithms are developed to synchronize data from the novel transducer topologies. A key component of the methodology is the acquisition of raw radiofrequency data. Unlike standard ultrasound imagery, radiofrequency data preserve the full signal information, which is critical for subsequent tissue characterization and the development of deep learning-based detection algorithms.

A critical requirement for autonomous CUS is the replication of manual clinical compression maneuvers through automated hardware. To achieve this, the wearable integrates a novel tissue compression actuator based on silent piezoelectric micropump technology and an array of air bladders to perform automated CUS by inducing and monitoring vessel collapse through real-time image feedback. Beyond imaging, the actuator supports VOP by precisely modulating thigh pressure to obstruct venous return while maintaining arterial flow. Designed for multiday wear, the actuator ensures stable sensor-to-skin contact and silent operation for patient comfort.

### Machine learning for automated DVT detection based on CUS

Automated DVT detection builds on machine learning models and aims to support 3 different tasks: image quality grading, vascular structure labeling, and guided compressibility analysis.

To ensure that only high-quality data are used for image analysis, an image grading model aims to continuously rate image quality on a 5-class scale and provide immediate feedback to the system’s control logic. Simultaneously, an image labeling model identifies and annotates vascular structures, as well as pathologies that could otherwise lead to misdiagnosis.

The core of the detection engine is a custom convolutional neural network based on the U-Net architecture, optimized for resource-limited mobile and embedded hardware. The final layer produces raw pixel probabilities, which are post-processed into color-coded segmentation masks for the user. The training dataset is acquired via 2 multicenter clinical studies conducted in the project. The data are de-identified and processed for experts’ annotations and partitioned based on stratified random sampling to ensure a similar distribution in training and test sets.

### Electrical impedance plethysmography and LRR

The ThrombUS+ solution integrates electrical impedance plethysmography and LRR into the wearable to evaluate venous capacity and enhance the diagnostic accuracy of the automated CUS DVT detection. Venous blood volume changes are captured by monitoring electrical impedance variations via an electrode array embedded in a smart textile sock, which utilizes conductive fabrics and tetrapolar configurations to optimize signal sensitivity. To distinguish weak venous pulsations from dominant arterial signals, the system employs dual-frequency spectroscopy and cardiac-cycle synchronization gated by real-time heart cycle detection and motion data. Diagnostic accuracy is further enhanced through controlled physiological interventions, such as pneumatic stasis induced by an inflation cuff and hydrostatic pressure changes triggered during limb elevation. Signal analysis is performed employing a machine learning pipeline that combines traditional classifiers like support vector machines with deep learning architectures such as long short-term memory and one-dimensional transformer networks. This regression-based approach provides a continuous estimate of thrombosis stages, facilitating the monitoring of thrombolytic treatment while compensating for movement artifacts. In parallel, miniaturized LRR sensors measure skin-level blood volume changes to proxy deep venous flow, with all sensors integrated into a low-power, wireless garment designed for point-of-care automation.

### Posture and physical activity tracking

Lower limb spatial orientation needs to be known before the initiation of DVT tests based on bioimpedance or light plethysmography. Additionally, monitoring of physical activity should be performed during the active DVT test and in between tests. Precise monitoring of physical movements will help filter/avoid movement artifacts and minimize the number of false DVT alarms. To track spatial orientation and physical activity patterns of the limbs, a network of inertial units is established. Real-time signal processing algorithms and machine learning are employed to recognize patient activity patterns. Training datasets are collected via respective clinical studies within the project.

### Wearable design

The ThrombUS+ device is designed as a 2-part wearable, comprising a strap for CUS and a smart sock or legging for comprehensive physiological monitoring. By embedding patented textile electrodes, strain gauges, and photonic sensors directly into the garment, the system ensures standardized sensor placement and reduces the workload for healthcare personnel. The design process considers a variety of natural and synthetic fibers that accommodate different genders, sizes, and specific clinical conditions such as limb lesions. Furthermore, the architecture adheres to the EU Strategy for Sustainable and Circular Textiles [37] through a modular construction that allows conductive yarns and sensing circuits to be easily separated from the textile base. This design choice extends the product life cycle by allowing for individual component replacement and ensures that the garment and electronic elements can be effectively recycled at the end of their life.

### Decision support and patient empowerment

The hardware is driven by a smart control software that incorporates a dual-interface decision support module designed to translate complex physiological data into actionable insights for both clinicians and patients. This layer integrates multimodal data, including ultrasound imaging, bioimpedance signals, and activity tracking, to provide diagnostic alerts and therapeutic guidance.

The clinician-facing application provides granular visual analytics and detailed diagnostic evidence to support complex medical decisions and allows the adjustment of treatment plans remotely, ensuring a continuous and optimized recovery process. The patient application employs simplified, age- and gender-sensitive interfaces to communicate DVT risk and guide preventive actions, prioritizing comprehension and the mitigation of potential anxiety or misinterpretation.

To address the high risk of DVT in bedridden or immobilized patients, the methodology features an XR and SG platform. This system enables physiotherapists to design personalized clinical exercise plans for rehabilitation and mechanical thromboprophylaxis. Patients engage with these plans through an immersive 3-dimensional (3D) environment, which provides real-time motion guidance by superimposing prescribed movements over the patient’s actual limb activity. A serious game component utilizes diverse, culturally respectful storylines to motivate patients, potentially including collaborative scenarios to foster a supportive community among patients with similar conditions.

### Implementation methodology

The implementation methodology follows the V-model [38], which establishes system implementation as an iterative, rather than linear process. Initially, based on human-centered design, a requirements analysis was conducted from which system specifications were identified and systems and subsystems were designed. During development and integration, the various modules were built and integrated. The last step includes testing modules and clinical validation of the entire system. In parallel, applicable legal and compliance requirements are addressed and applied throughout all the aforementioned steps, ensuring a compliance-by-design approach. Furthermore, from the very outset of the project, additional activities, such as dissemination, communication, exploitation, and commercialization, accompany its progress to maximize the dissemination of outputs and explore potential future market directions. Figure 2 depicts the implementation process of the ThrombUS+ system.

**Fig. 2.**
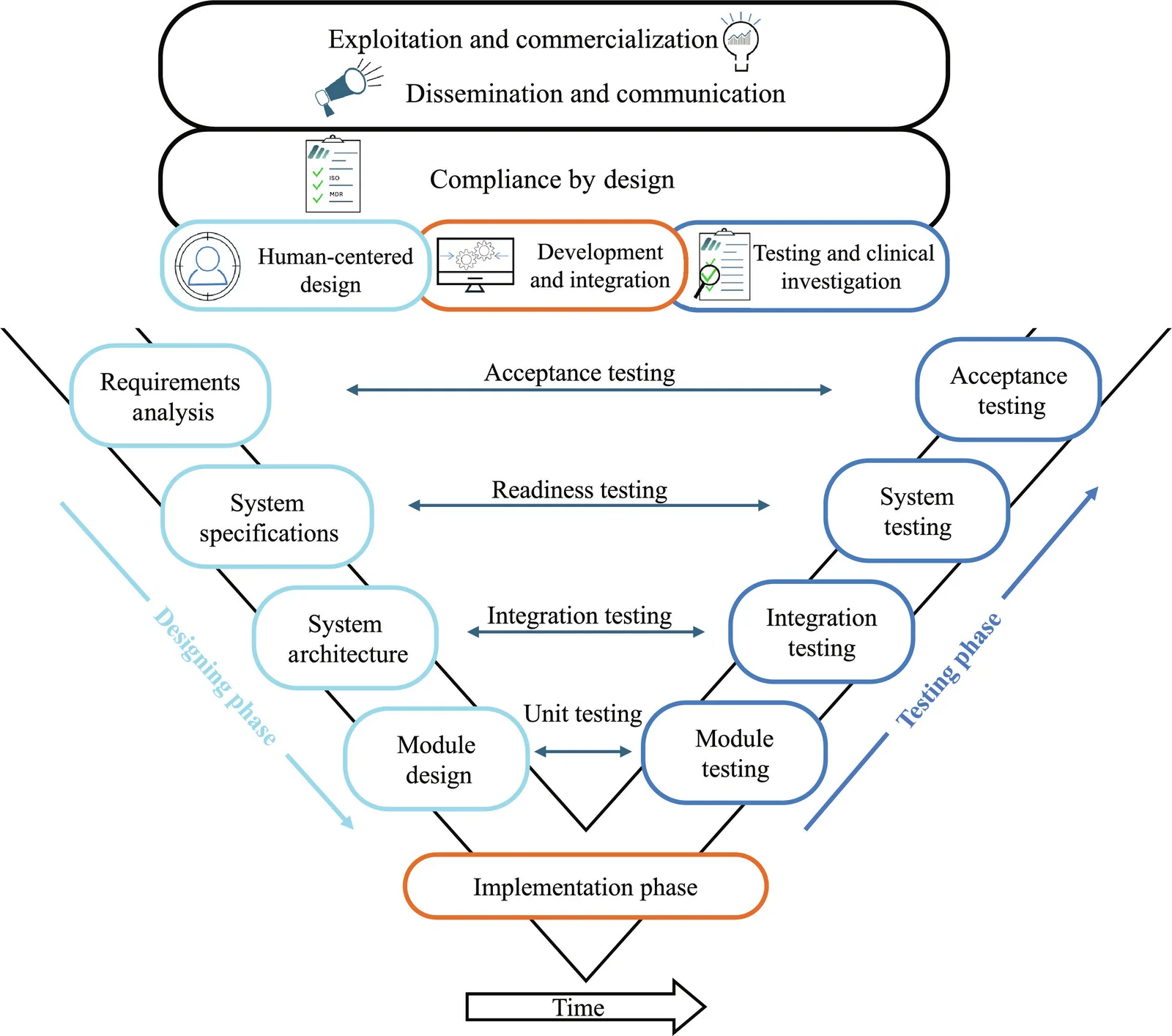
High-level implementation methodology of the ThrombUS+ project.

#### Requirements analysis

The requirements analysis and definition process follows a human-centered design approach [39], leveraging contextual understanding and early, continuous stakeholder engagement. Importantly, in this context, stakeholders include not only the direct end-users involved in the co-design process, but also anyone affected by the introduction of the system.

To this end, a combination of data collection and analytical methods was employed, including an exploratory literature review, consultations with clinical and technical experts through interviews, and the administration of targeted questionnaires [40]. These activities aimed to (a) profile different types of patients based on their clinical conditions and usage contexts; and (b) identify and prioritize deployment scenarios where the ThrombUS+ solution is expected to have maximum impact, based on an analysis of epidemiological data and unmet practical needs of DVT early detection and management.

Data from the literature review on DVT risk factors and insights from clinical experts gathered via structured interviews were analyzed and combined to define relevant patient profiles. These profiles include clinical contexts, patient journeys, and functional design requirements for a usable, acceptable, and cost-effective device for automated continuous monitoring of DVT. Patient personas (plain-language narratives illustrating typical users’ journeys) were created to help developers and stakeholders understand user needs and support design decisions.

#### Regulatory and compliance by design aspects

The ThrombUS+ project adopts a compliance-by-design approach, meaning that applicable legal and regulatory requirements are addressed from the very beginning and systematically integrated into all phases of the design and development process [41]. This procedure ensures ongoing regulatory alignment with European legislation.

The overall system developed within the ThrombUS+ project qualifies as a medical device and is therefore subject to the regulatory requirements of the Regulation (EU) 2017/745 on medical devices (MDR). In accordance with the MDR, the strategy for regulatory compliance encompasses processes for the identification of relevant legal requirements, qualification, classification, handling of equivalence, choice of, and compliance with conformity assessment procedures. The starting point for fulfilling the regulatory requirements under MDR was defining the intended purpose and determining the MDR risk class. Although the product will not be placed on the market within the scope of this project, an outlook on possible post-market surveillance activities will nevertheless be provided in the future.

In addition, because of the integration of AI-based subsystems intended to support the monitoring of DVT development, the system also falls within the scope of the Regulation (EU) 2024/1689 on AI (AI Act [AIA]), which introduces specific requirements for AI systems in Europe. According to Art. 6 (1) AIA, the ThrombUS+ system qualifies as a high-risk AI system. Consequently, it must comply with the provider requirements set out in Art. 16 AIA and undergo a conformity assessment by a Notified Body. Central elements of the conformity assessment include the technical documentation for the AI systems and the related quality management system.

#### Integration and testing methodology

Integration and testing follow a comprehensive systems engineering approach that addresses both technical integration challenges and clinical validation requirements. The integration phase encompasses the interconnection of the ThrombUS+ sensing modalities, which require sophisticated data fusion algorithms and real-time processing capabilities to synthesize multimodal physiological signals into clinically actionable information.

The testing methodology adopts a phased validation approach, beginning with individual component testing to verify sensor accuracy and actuator performance under controlled laboratory conditions, followed by integrated system testing to evaluate intermodule communication. System readiness testing involves a comprehensive evaluation of the entire integrated ThrombUS+ device using the Central Hub and Intelligence (CHI) unit, replicating real-world working modes. Beyond functional requirements, this phase rigorously assesses user safety, information security, data privacy, connection stability, and overall system robustness and reliability. Successful completion of these tests signifies that the system meets all user and system requirements, is safe and reliable, and is ready for progression to human clinical trials. Clinical validation involves controlled studies comparing the ThrombUS+ system against the gold standard duplex ultrasonography, incorporating both healthy volunteers and confirmed DVT patients to establish sensitivity and specificity thresholds for automated detection across diverse demographic groups. Usability testing with healthcare professionals and patients ensures that the operator-independent design meets clinical workflow requirements and user acceptance standards. The iterative V-model approach allows continuous refinement of integration parameters and testing protocols based on preliminary results, ensuring the final system meets both technical specifications and clinical efficacy standards.

#### Clinical studies

The project research and innovation heavily rely on several prospective clinical studies to collect required training data based on robust protocols. These datasets are essential for developing and training the machine learning or deep learning models used by the ThrombUS+ device. All studies adhere to GDPR/EHDS/MDR regulations, ensuring complete anonymization and overall regulatory compliance. Data collection studies, named clinical study A and B1 to B4, will be followed by studies evaluating the feasibility of the entire system.

Clinical study A aims to collect labeled ultrasound images from 3,000 patients suspected of DVT who are undergoing routine CUS scans [42]. Clinical study B1 uses the novel ultrasound system developed in the project to manually scan patients suspected of DVT. The collected and curated dataset will be used to explicitly fine-tune the AI models previously developed using images from clinical study A.

Clinical studies B2 and B3 will use commercial and prototype inertial measurement units, respectively, to collect activity data from healthy individuals during physiotherapy exercises, to be used in training machine learning models for posture and activity recognition. Finally, in clinical study B4, impedance plethysmography data will be collected from healthy volunteers and patients diagnosed with DVT and referred to venous thrombolysis treatment. The collected and curated data will be used to develop signal processing methods and machine learning models for impedance-plethysmography-based diagnosis of DVT. In addition, the anonymized data will be made publicly available by publishing it in the European Open Science Cloud.

Acceptance testing of the integrated ThrombUS+ device is planned via 2 clinical trials. First, an early feasibility clinical study aims to assess device design, safety, patient acceptance, and functionality with the involvement of 50 to 100 bedridden patients within the participating hospitals. The second clinical trial comprises a controlled, double-blind trial comparing the wearable device against conventional ultrasound DVT scanning, with sample size calculations targeting 150 to 200 patients.

### ThrombUS+ consortium

Taken together, the project’s complexity requires collaboration among professionals from diverse backgrounds and expertise. The consortium brings together academia, research institutes, industry, and hospitals, integrating disciplines across various fields of health and health sciences. Beyond technological expertise, consortium members bring extensive experience in human sciences and socioeconomic research, healthcare provision, regulatory and compliance affairs, and dissemination and communication. In total, 18 partners from Greece, Lithuania, France, Germany, Italy, Finland, Spain, and the United States participate in the project (Fig. 3).

**Fig. 3.**
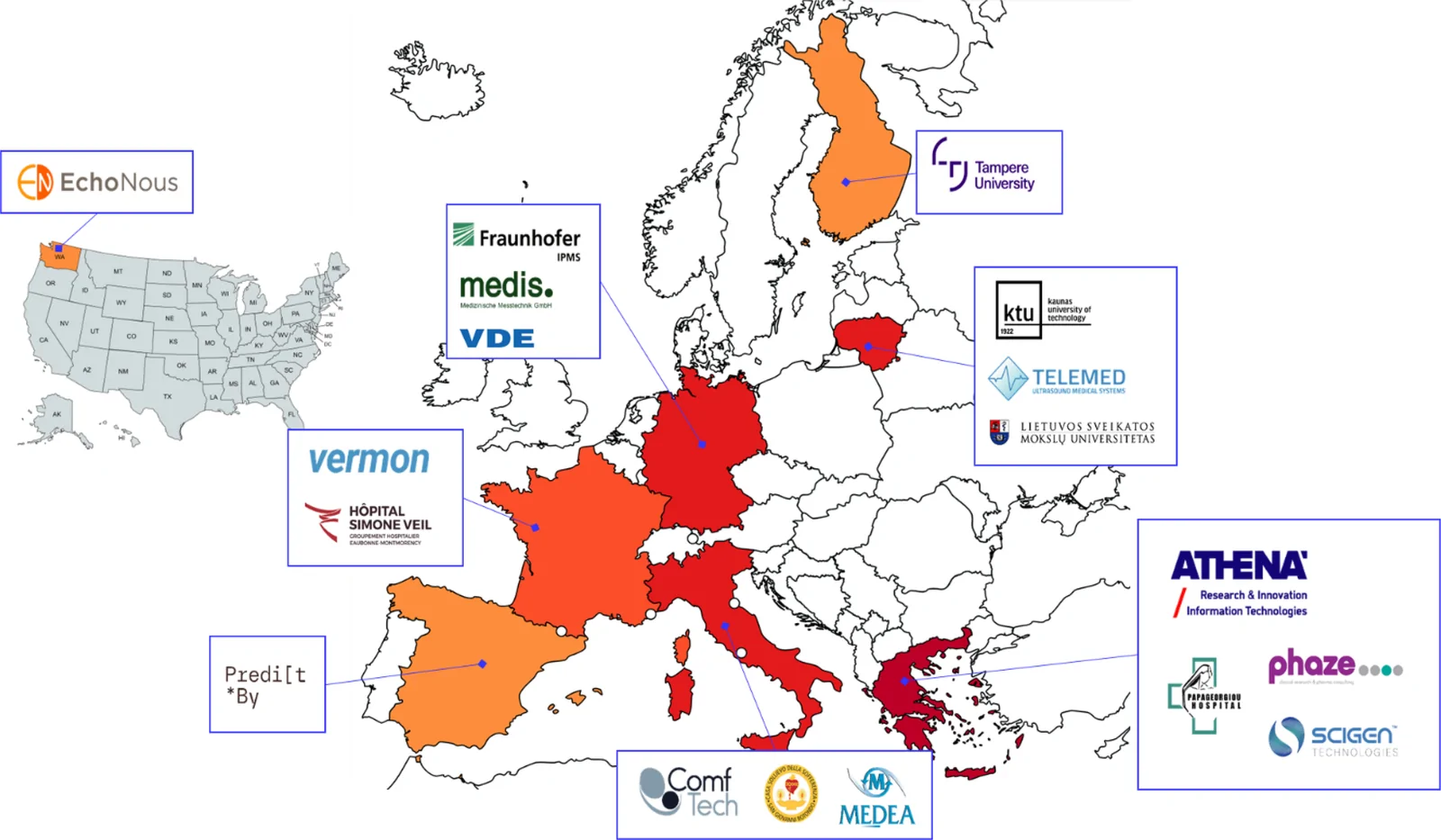
The ThrombUS+ consortium comprises multidisciplinary and multinational partners across Europe and the United States.

Technological partners Vermon and Fraunhofer IPMS provide high-quality ultrasound transducers tailored to system requirements. TELEMED develops compact and energy-efficient electronics for ultrasound image formation, while EchoNous elaborates on machine learning models for automated ultrasound image assessment. Tampere University (TAU) and Medis develop complementary DVT-detection sensors using electrical impedance plethysmography and LRR, respectively. Kaunas University of Technology develops the wearable actuator for operatorless CUS and VOP, along with lower-limb activity sensors to be integrated into ComfTech’s smart garments, and also leads prototype testing and hardware integration. ATHENA RC, as a computational science and technology expert, leads software development and integration. ATHENA RC will develop an XR environment and a SG software for DVT prevention. The software will use lower-limb activity data to guide and motivate patients in performing DVT-preventive physiotherapy exercises. Also, ATHENA RC is responsible for developing the ThrombUS+ application, an intelligence and decision-making application that will orchestrate the overall system’s functionality at the software level.

Clinical partners TAU, Lithuanian University of Health Sciences, General Hospital Papageorgiou, Fondazione Casa Sollievo Della Sofferenza, and Groupement Hospitalier Eaubonne Montmorency Simone Veil, assisted by the General University Hospital of Alexandroupolis (which has joined the consortium as an Associated Partner), primarily contribute to the implementation of all clinical studies. The contract research organization PHAZE coordinates and supports all clinical activities, ensuring compliance with both international and national regulations. PredictBy, with social science expertise, leads the user requirements definition for the system and conducts cost-effective analysis of the final solution. VDE contributes its expertise to identify regulatory requirements and ensure regulatory compliance across all aspects of the project. Medea leads exploitation and commercialization activities, while SciGen leads the communication and dissemination activities. ATHENA RC coordinates the project by overseeing all ongoing activities and ensuring seamless, robust collaboration and communication among partners. The partners forming the ThrombUS+ consortium can be found on the project website https://thrombus.eu/ or https://ec.europa.eu/info/funding-tenders/opportunities/portal/screen/opportunities/projects-details/43108390/101137227.

The total project budget is €9,565,763.75 and is co-funded by the European Union Horizon Europe Innovation Action (HORIZON-HLTH-2023-TOOL-05-05) by €8,141,251.25. Project agreement number is 101137227.

## Preliminary Results and Impact

At this stage of the project, the design phase is complete, and the development phase is underway, with the hardware components fully developed and tested. Software development is underway, and integration has been initiated. Data collection clinical studies are well underway, while the early feasibility study and the clinical trial are currently being designed.

### Identification of intended purpose and use cases

For regulatory classification and approval, the intended purpose of the ThrombUS+ device was defined as follows: “ThrombUS+ is an active, wearable diagnostic device for point-of-care, continuous (intermittent) monitoring of patients at increased risk of DVT, which is applied to the patient’s lower limb. For this purpose, ThrombUS+ autonomously combines compression ultrasound (CUS) imaging, electrical impedance plethysmography (EIP), and light reflection rheography (LRR) to assess blood clot formation and blood flow on the lower limbs. Additionally, ThrombUS+ uses inertial measurement units (IMUs) to monitor patients’ lower limb activity. Via an extended reality (XR) environment and serious gaming (SG), patients are trained and motivated, respectively, to perform lower limb exercises for DVT prevention at the point of care. ThrombUS+ is intended for application to adult patients in the clinical environment by healthcare professionals”. The term “continuous (intermittent) monitoring” denotes longitudinal monitoring over extended periods, where measurements are acquired at predefined or adaptive intervals rather than continuously, enabling efficient and patient-friendly assessment of DVT risk. Based on this intended purpose, the ThrombUS+ system is expected to be assigned to MDR Risk Class IIb [43].

The patient profiling exercise identified 80 patient profiles and 91 supporting bibliographic references. The identified profiles were then cross-analyzed with the findings of the literature review on concurrent conditions, followed by a systematic grouping and prioritization exercise. It was noted that several of the identified profiles were either highly specific (e.g., severe traumatic brain injury with neurological deficits) or showed substantial overlap in the clinical approach and the potential added value of a device such as the ThrombUS+ (e.g., hip, knee, or pelvic surgery). Following an intensive prioritization and clustering process—based on prevalence, DVT likelihood, DVT incidence, and risk ratio scores—7 main reference use cases were identified:

1. neurosurgery;
2. lower limb orthopedic surgery;
3. cardiovascular surgery and risk;
4. pregnancy and postpartum;
5. obesity;
6. cancer and oncological treatment; and
7. autoimmune diseases and genetic disorders.

From the identified reference use cases, only post-surgery-related reference use cases (neuro-, lower-limb orthopedic, and cardiovascular) will be considered for the later stages of the project.

### High-level system architecture, requirements, and development of prototypes

The ThrombUS+ device comprises 4 subsystems for the autonomous monitoring and prevention of DVT. Each subsystem consists of a combination of modules, the building blocks of the entire device. Figure 4 presents a high-level overview of the system architecture, depicting the individual modules and how they are combined to form functional subsystems, which in turn integrate into the strap and legging wearable.

**Fig. 4.**
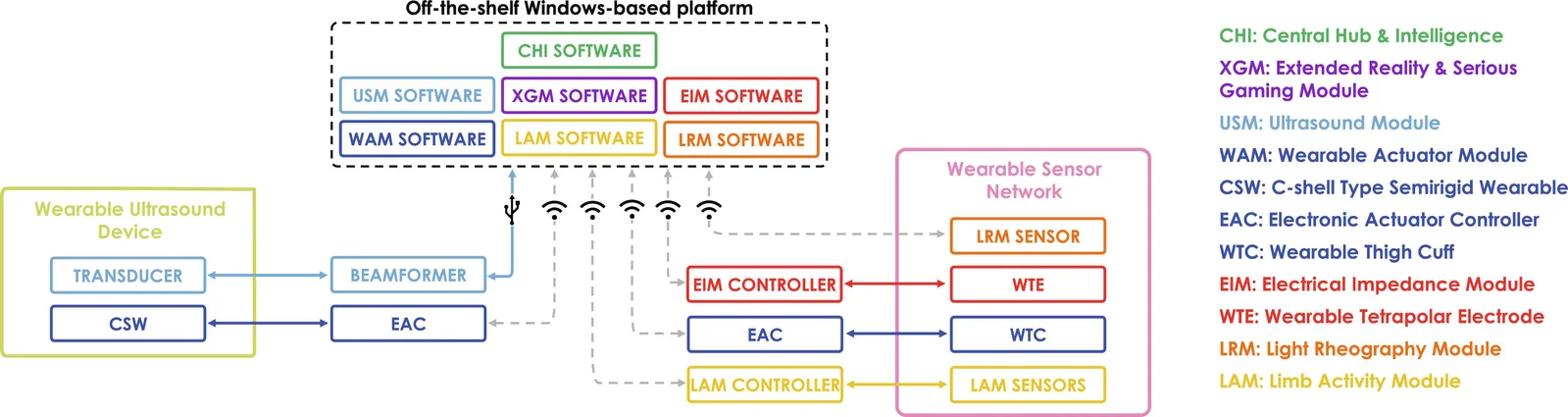
The high-level architectural design of the ThrombUS+ device. The device consists of individually functioning modules, which combine to form 4 subsystems that enable autonomous, continuous monitoring, and prevention of DVT. The Central Hub and Intelligence (CHI) module is the only pure software module, orchestrating the system’s functionality in a module-based manner.

Three subsystems implement complementary, operator-independent methods for continuous DVT monitoring, while the fourth subsystem enables DVT prevention methods. First, the combination of the wearable ultrasound system with the wearable actuator enables automated ultrasound compression. Second, the wearable actuator, together with the electrical impedance module, enables the performance of the VOP method to evaluate blood volume in the lower limbs. Third, by utilizing light rheography sensors, placed above the ankles, blood volume changes during ankle dorsiflexion can be measured. Finally, for DVT prevention, through an XR and SG application, patients are guided and motivated to perform DVT-preventive physiotherapy exercises. The CHI application, serving as the main ThrombUS+ application, coordinates all actions and devices and provides the user interface.

Four core user groups were identified: (a) medical specialists (doctors, nurses, and technicians); (b) patients; (c) maintenance engineers; and (d) biomedical researchers. A total of more than 80 different user requirements were identified and formally described. Based on these user requirements and taking into account the compliance by design approach, more than 100 system-level requirements were identified and formally described for the entire system.

### Wearable compression ultrasonography subsystem

The compression ultrasonography subsystem enables operator-independent DVT monitoring in high-risk patients using machine learning and deep learning for image processing and decision-making. Automating CUS allows prolonged, intermittent monitoring, especially after surgery. The subsystem combines an ultrasound imaging module, a wearable actuator for tissue compression, and a CHI module for coordination (Fig. 5A). Automatic compression is achieved by a pneumatic actuator using air bladders pressurized by a silent piezoelectric air pump. The CHI controls the actuator, intermittently compressing the lower leg while acquiring B-mode ultrasound images of vascular structures (Fig. 5B).

**Fig. 5.**
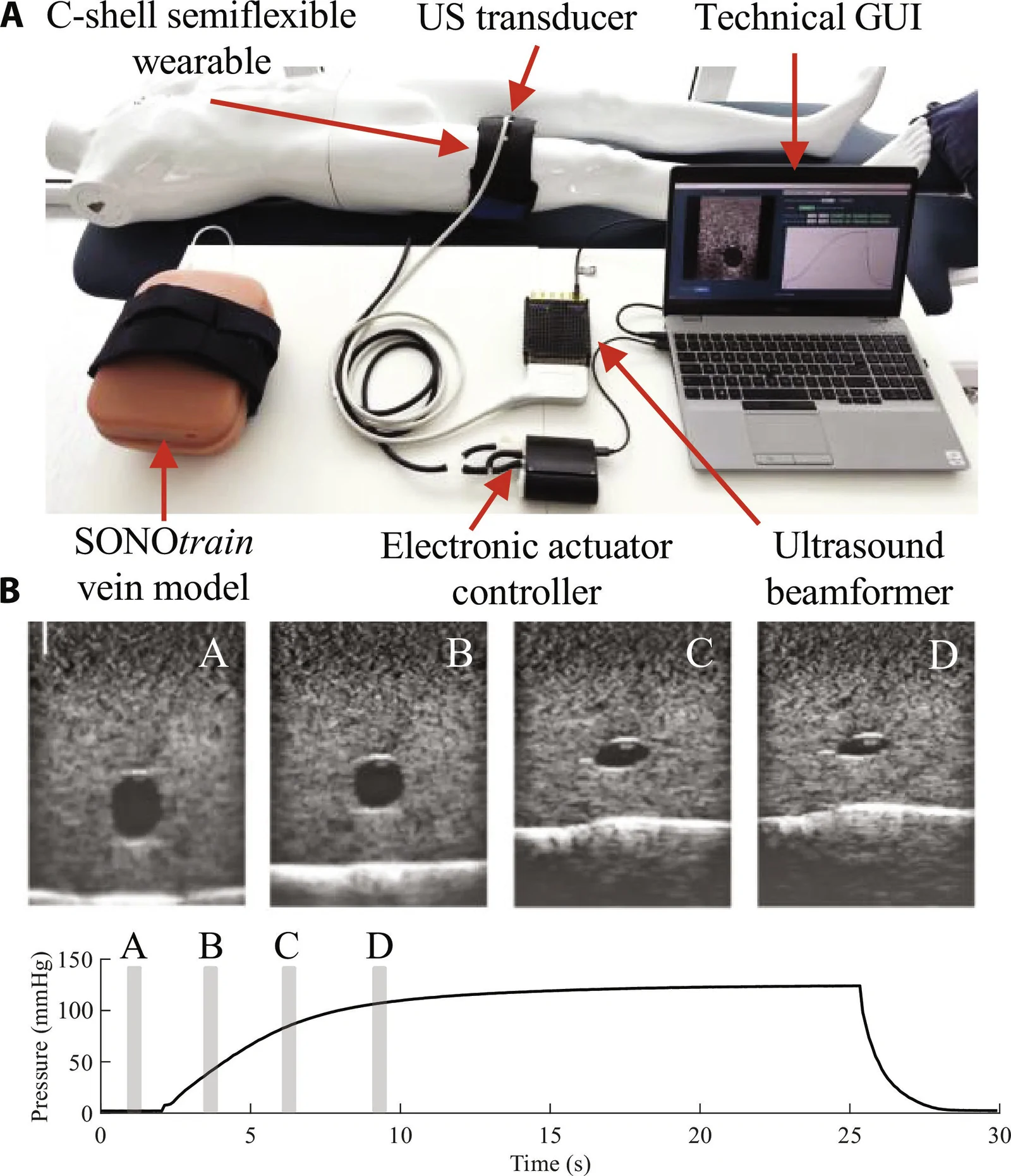
The ThrombUS+ compression ultrasonography module. (A) System configuration and experimental setup for laboratory testing. (B) Representative B-mode image sequence acquired during automated tissue compression of a tissue phantom. The wearable module integrates a linear ultrasound probe and an inflatable bladder for controlled compression, driven by a portable beamformer and a low-noise air pump. Experiments were performed using the SONOtrain Ultrasound Vein Model (3B Scientific GmbH, Germany).

### Wearable plethysmography subsystems

The VOP subsystem is designed to measure electrical impedance changes in the lower limb during and after occluding the thigh veins. To this end, this subsystem utilizes the electrical impedance module [44] and the wearable tissue compression module [45] (Fig. 6). The electrical impedance module integrates novel wearable textile electrodes in a tetrapolar configuration and a portable controller to deliver and measure high-frequency, low-amplitude current, while the wearable tissue actuator applies tissue compression to occlude blood outflow and releases it, as required. The measured electrical impedance can be correlated with blood volume changes and, via machine learning and deep learning signal processing methods, used to assess the presence of a physiological or pathological condition.

**Fig. 6.**
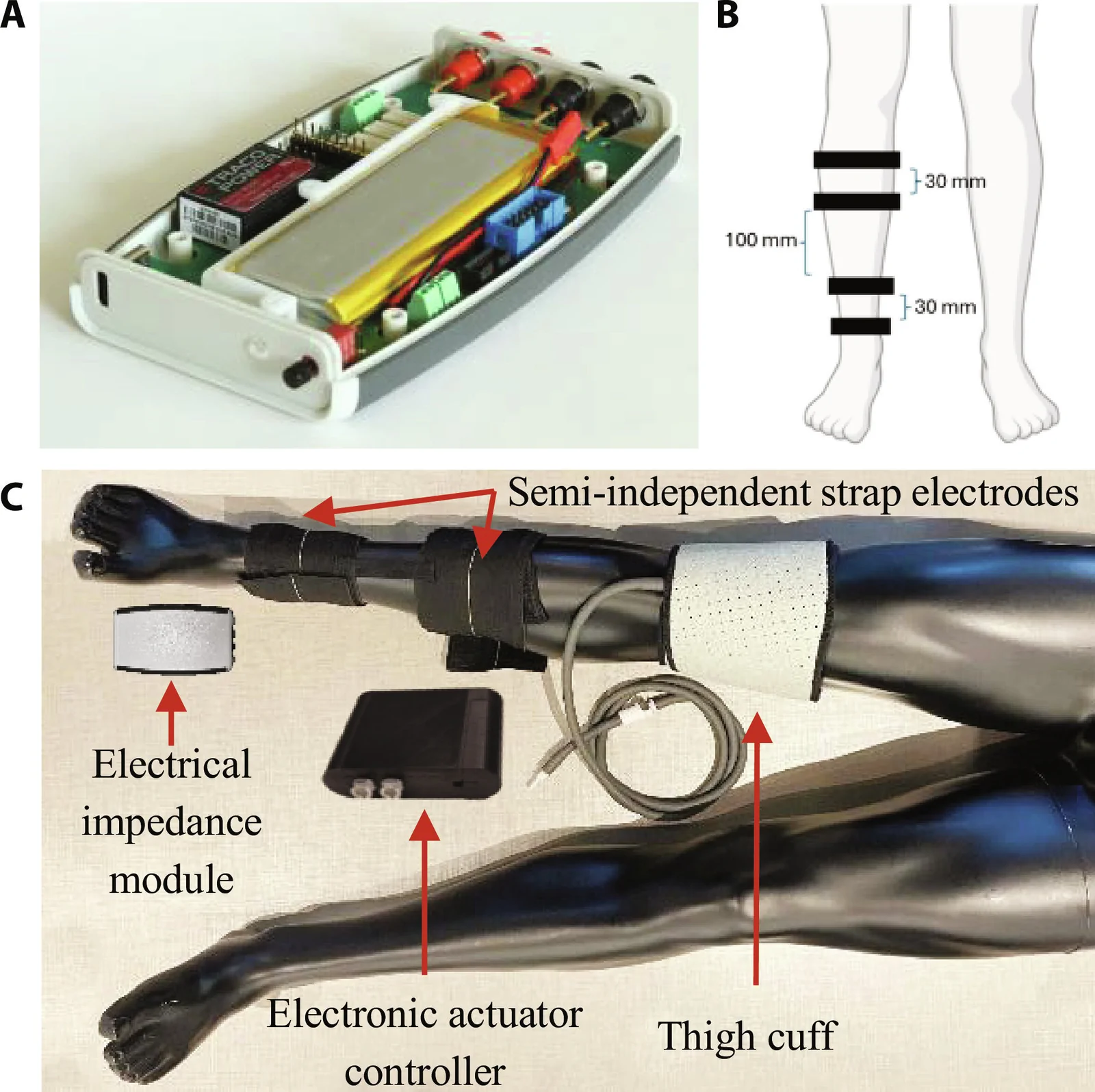
Electrical impedance plethysmography module for implementing the venous occlusion plethysmography method. (A) The controller of the electrical impedance module integrates a front-end circuit, a digital signal processor, and a Wi-Fi communication capability, and is powered by a rechargeable battery via USB. (B) Selected wearable electrode, placed in a tetrapolar setup. (C) The overall subsystem, including the electrical impedance and the wearable tissue actuator modules.

The electrical impedance controller is implemented using an analogue front-end circuit with a digital signal processor to facilitate the integration of impedance signal processing and DVT detection algorithms. Communication between the controller and the CHI module is established via a Wi-Fi connection. The module is powered by an integrated rechargeable battery, thereby making the device totally portable and reusable.

Several wearable electrodes were developed and tested. The optimized electrode setup was chosen based on electrical impedance plethysmography and spectroscopy of the various preliminary setups. Based on the optimization process, a semi-independent strap electrode provided the best combination of signal quality, usability, and comfort.

Furthermore, a novel ultraportable wearable sensor has been developed to allow DVT assessment via light reflection (Fig. 7). The sensor is placed above the ankle and emits infrared light to analyze the photometric properties of the superficial tissue by measuring the amount of absorbed infrared light by hemoglobin in the veins and arteries. Based on these measurements, the blood volume changes in the superficial tissue are estimated during a tiptoe exercise, and the results are correlated with the presence of physiological or pathological condition.

**Fig. 7.**
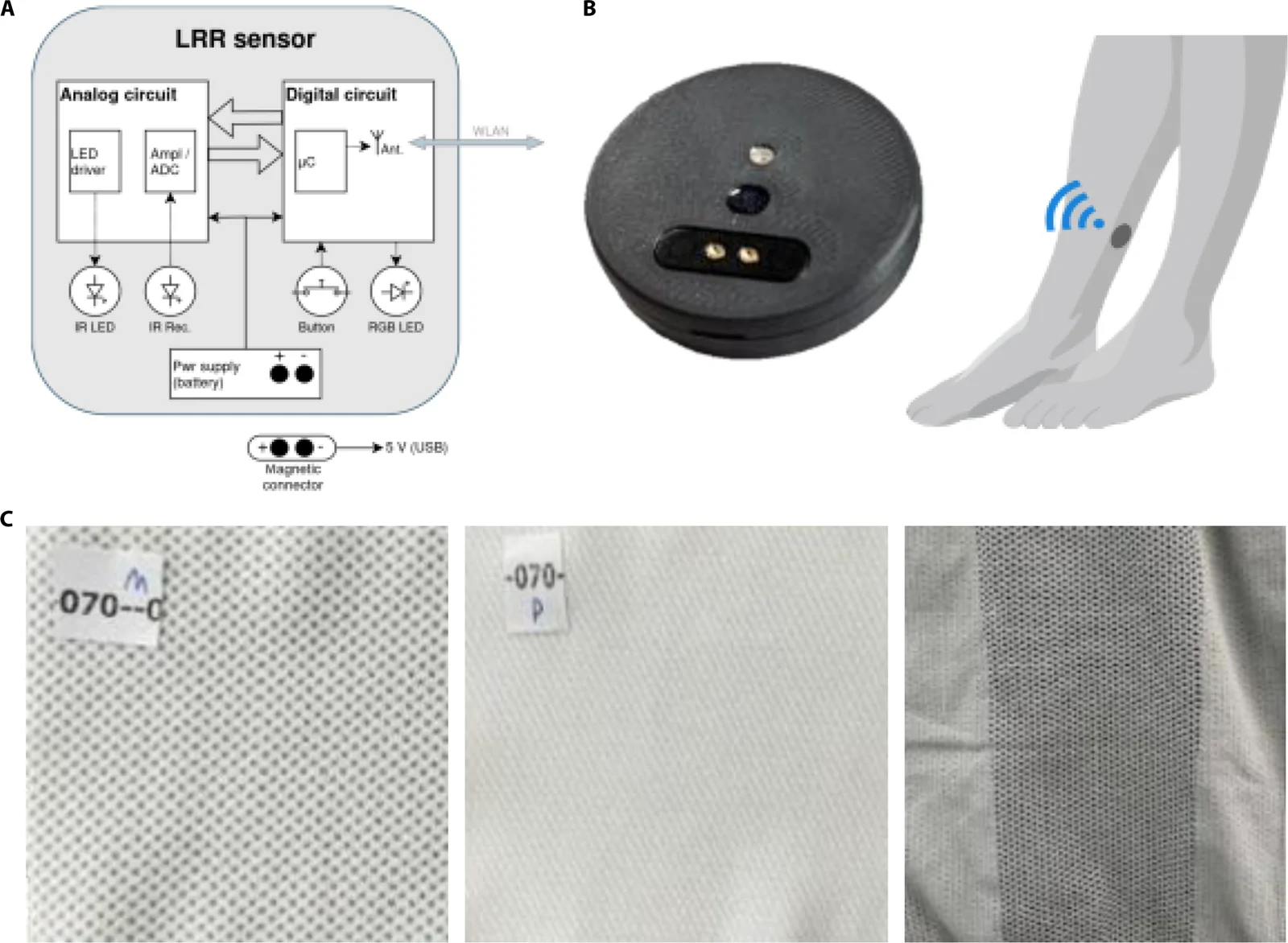
Light reflection module. (A) The sensor architecture design depicts the hardware elements. (B) The first assembled prototype sensor and the proposed placement of the lower limb. (C) The biocompatible fabrics were tested for their optical properties. The fabric on the left and the middle proved suitable for integrating the light reflection sensor into the final wearable.

The sensor has the size of a button and consists of an infrared light emitter and receiver, a digital and an analogue circuit to enable on-device signal processing and feature extraction, and a Wi-Fi antenna for communication and data transfer with the CHI module [46]. The sensor is powered by an integrated rechargeable battery, making it ultra-portable and reusable. This design allows the sensor to be fully integrated into the final textile, without the need for extra cables.

Future work focuses on testing, improving, and miniaturizing electrical impedance and light-reflection sensors, as well as enhancing signal quality. In addition, using clinical data acquired through deployed clinical studies will drive the testing and development of novel signal-processing algorithms and AI-based models for operator-independent plethysmography-based assessment of DVT.

### Multimodal monitoring

The ThrombUS+ system provides longitudinal multimodal monitoring over extended periods, utilizing measurements sampled at predefined or adaptive intervals. As no established framework currently exists for synchronized fusion of ultrasound, electrical impedance plethysmography, and LRR, our approach focuses on the complementary strengths of these modalities. Data from each sensing stream is processed via a modality-specific pipeline to extract physiologically meaningful features. The system is designed for high resilience. Depending on patient-specific constraints, such as wound locations, contraindicating ultrasound, or mobility issues preventing LRR-specific exercises, the architecture adapts its monitoring strategy to the available data.

A primary objective of the upcoming clinical studies is to characterize the diagnostic synergy between localized imaging (CUS) and global hemodynamic markers. While compression ultrasonography provides high sensitivity and accuracy at detecting thrombus at the specific anatomical site of the wearable, typically at the femoral vein, it may not detect isolated proximal or distal thrombi. In contrast, VOP captures integrated hemodynamic changes reflecting venous outflow and refill across the entire lower limb. Because VOP electrodes, placed around the knee, and LRR sensors, placed more distally (above the ankle), monitor blood pooling and clearance influenced by the entire venous network, they may offer proxy detection for clots outside the CUS field of view. Consequently, the later-stage ThrombUS+ clinical trials will investigate the optimal fusion logic required to bridge these modalities, determining how global hemodynamic anomalies from LRR/VOP can complement localized CUS data to achieve comprehensive longitudinal DVT surveillance.

Practical deployment considerations were also evaluated for the proposed ThrombUS+ system. As the platform integrates several diagnostic and prophylactic subsystems, operational parameters depend on the specific modality used. Monitoring cycles are designed to operate periodically, with measurement duration and frequency determined by the selected diagnostic test and clinical use case. Patient positioning requirements vary across modalities (e.g., compression ultrasonography may be performed while lying in bed, VOP in a supine position with elevated legs, and LRR in a sitting position). Certain subsystems, such as the compression ultrasonography actuator, are designed to be lightweight and silent, enabling continuous wear and potential overnight use, whereas other functions requiring patient interaction are intended for awake operation. Because of physiological variability between individuals, patient-specific calibration is required for most sensing subsystems. As the system is currently under development, battery life and long-term usability will be evaluated during the upcoming prototype and clinical validation studies.

### SG and XR environment for patient empowerment

In addition to ultrasound- and plethysmography-based subsystems for DVT detection, the ThrombUS+ device also incorporates a DVT-prevention subsystem. The subsystem is designed to promote patient empowerment and adherence to physiotherapy exercises, enhancing mobility and reducing the risk of DVT. The DVT-prevention subsystem combines the limb activity module for tracking lower limb kinematics, enabling engagement in various SG scenarios through active movement of the lower limbs as a means of interaction, and the visualization of the lower limb kinematics in an extended environment for training, real-time guidance, and exercise assessment.

For precise tracking of lower limb activity, the limb activity module combines 6 inertial measurement units integrated into the wearable textile, positioned on the thigh, shin, and foot of each leg (Fig. 8). These sensors measure inertial signals and calculate the orientation of the respective leg segment they are attached to. Limb activity sensors also integrate photoplethysmography and temperature sensors for future multimodal model development, incorporating leg segment-specific activity and physiological measurements.

**Fig. 8.**
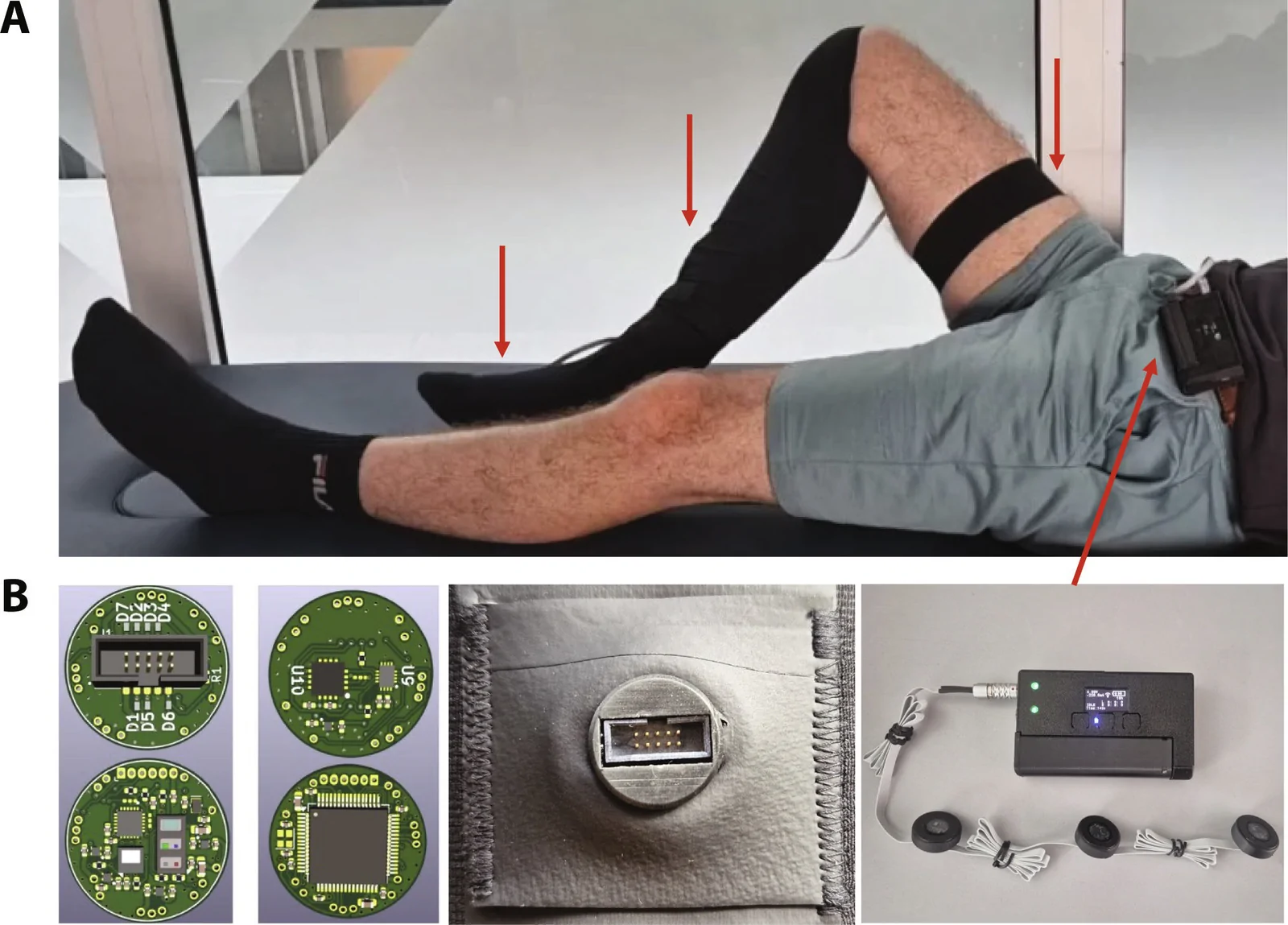
The limb activity module for tracking lower-limb kinematics using inertial measurements. (A) A prototype wearable integrating 3 activity sensors positioned on the right thigh, shin, and foot. (B) From left to right: single sensor printed circuit board rendering (20 mm in diameter); indicative sensor integration within the wearable; and integrated sensors and controller for powering and controlling up to 7 sensors.

The XR environment facilitates patient training in performing the prescribed exercises correctly by enabling the visualization of reference physiotherapy exercises in a realistic, interactive 3D environment (Fig. 9A). Furthermore, the XR environment will enhance treatment efficacy by comparing the patient’s current position with the reference posture, enabling real-time guidance and feedback (Fig. 9B and C). On the other hand, serious games are employed to enhance patient empowerment in performing physiotherapy exercises and to increase mobilization of the lower limbs through a more engaging approach. Various serious game storylines are adapted, each using a different lower limb exercise group to drive the game and thus address a broad spectrum of patient needs (Fig. 9D).

**Fig. 9.**
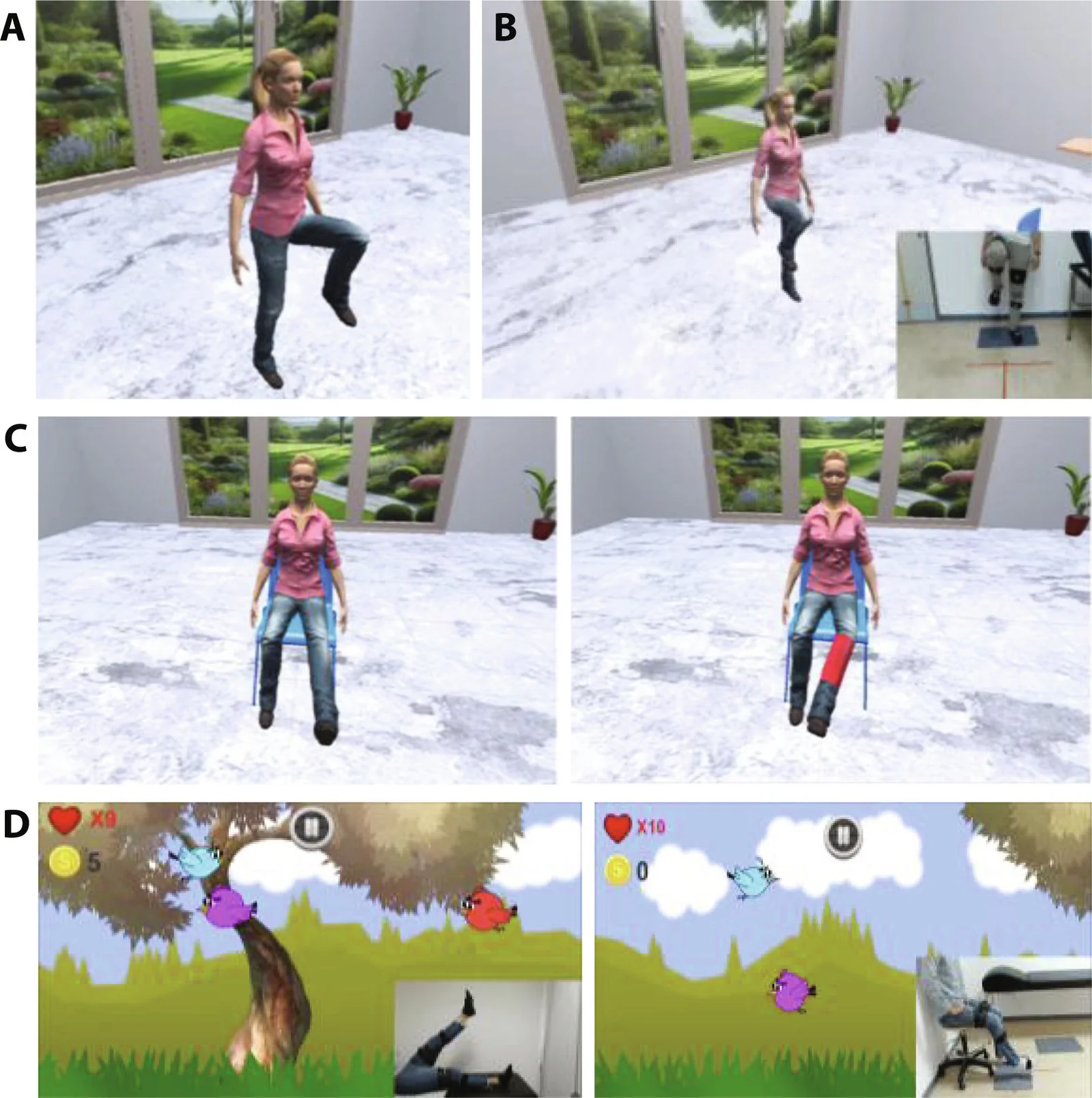
Extended reality (XR) and serious gaming module. (A) The XR environment enables interactive 3D visualization of reference physiotherapy exercises in both spatial and temporal dimensions. (B) Using sensor data from the limb activity module, the patient’s current posture can be visualized in real time during physiotherapy exercises. (C) Real-time comparison of the current posture with the reference posture provides visual feedback and guidance, with red coloring indicating deviations from the reference movement. (D) In the serious gaming implementation, the position of a bluebird character is controlled by sensor data from specific leg segments.

### Central hub and intelligence

The CHI module orchestrates the integration of the device’s modules at the software level, providing the tools, methods, and intelligence to set up the ThrombUS+ device and enable its autonomous operation in the clinical environment. The application is structured into 2 levels: backend services and the graphical user interface (GUI). The backend services encompass the core services of the application, including logging, database-specific services, and model runtime services that manage the device’s overall operation. Additionally, the backend services include software development kit services that are responsible for communication and control of the device’s hardware modules (Fig. 10). On the other hand, the GUI window handles all the graphical components essential for user-application interaction.

**Fig. 10.**
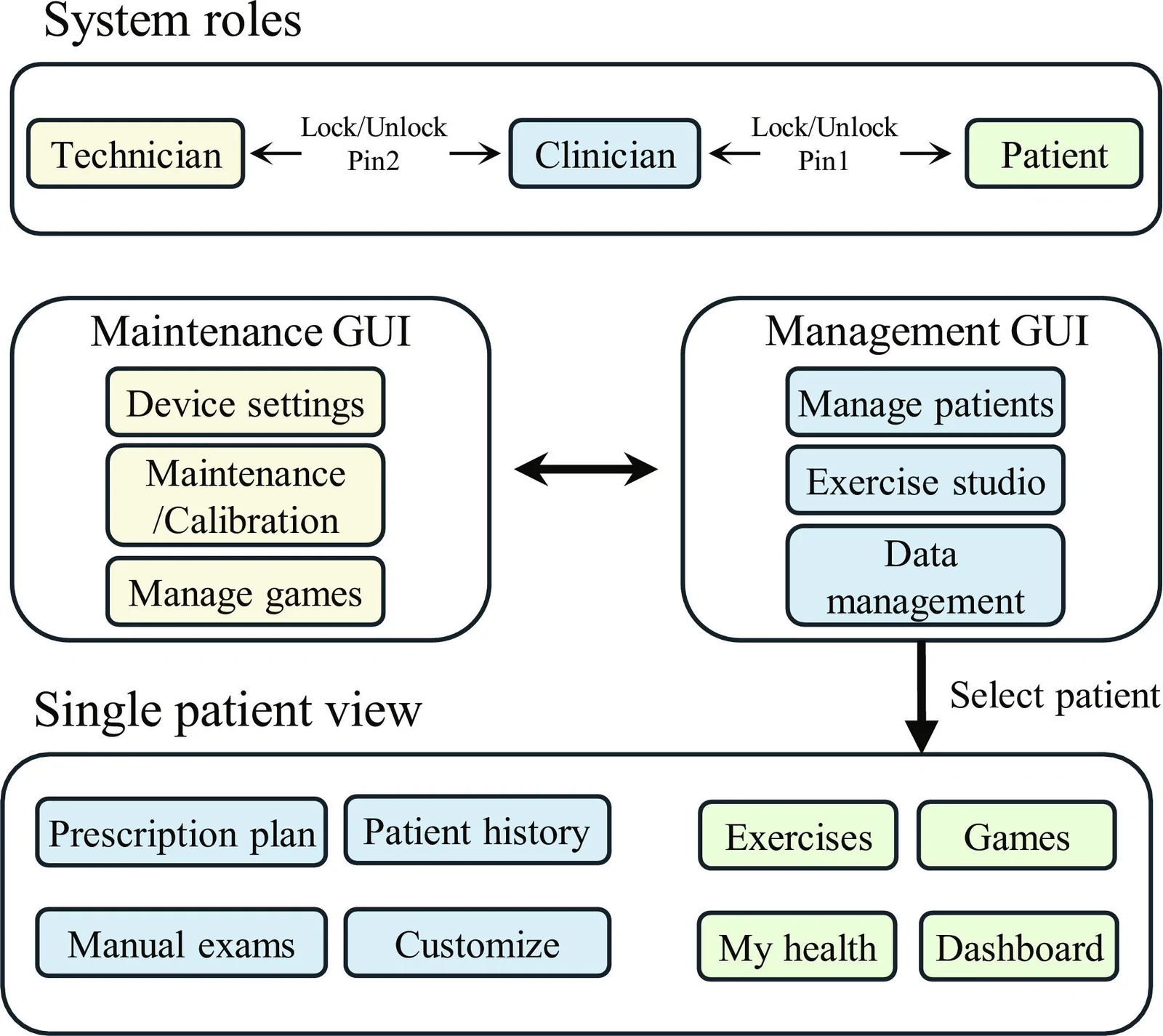
Structure of the graphical user interface. The system supports 3 distinct user roles—technical, clinician, and patient—each with role-based access control to ensure secure use of the platform. For each role, the interface provides tailored functionalities aligned with the specific tasks, workflows, and routines required.

More specifically, the GUI (Fig. 11) allows user-application interaction at 3 levels based on the user’s role. Switching between roles requires a personal identification process. At the technical level, the interface allows access to the device’s settings for maintaining, updating, and managing the device at a low level. At the clinical role level, the interface allows patient management, patient-related data management, management of reference physiotherapy exercises, and on-demand manual DVT assessment using device modules. Finally, at the patient level, only patient-specific data can be accessed and managed. This includes setting up the patient’s health record, the patient’s physiotherapy prescription plan, and the patient’s DVT autonomous monitoring plan. This role also enables the autonomous operation of the device according to the prescribed plan.

**Fig. 11.**
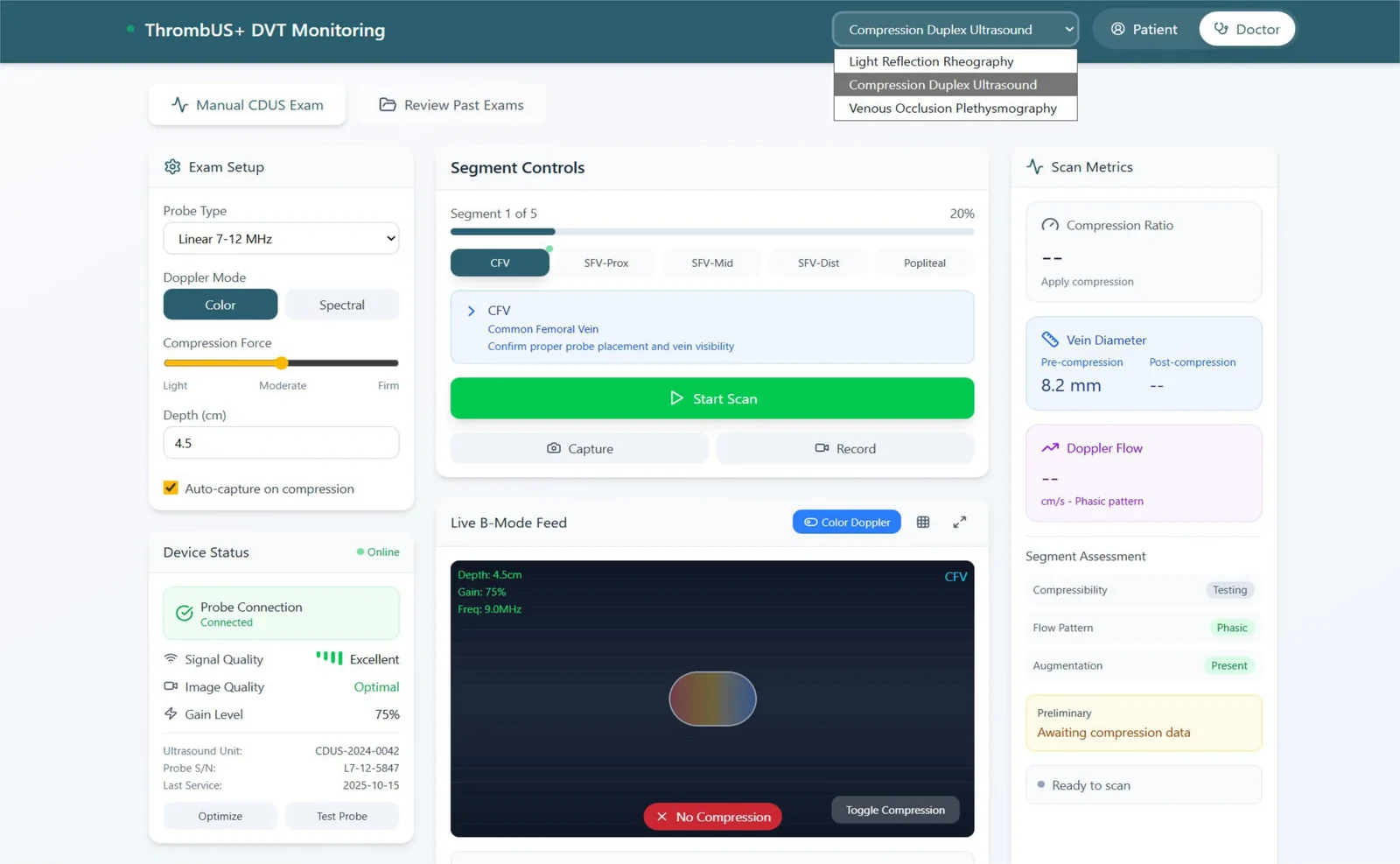
Exemplary graphical user interface (GUI) of the CHI module. The interface provides role-based access for the Patient and Doctor sections. The panel shows the CUS testing window in the doctor section, with the capability to perform additional tests (light reflection rheography [LRR] and venous occlusion plethysmography [VOP]).

During autonomous operation, the user interface allows navigation through different DVT monitoring methods to observe examination progress in real time or visualize examination records. Finally, the patient can engage in XR or SG if no DVT examination is in progress.

### Risk and failure modes management

Potential risks associated with both hardware and software components of the ThrombUS+ system were considered from the early stages of design and development. Risk management is conducted in accordance with EN ISO 14971, encompassing the identification, analysis, and mitigation of safety and security risks, as well as their interactions. This framework also addresses risks related to the implementation of AI, including the potential for performance drift, which is managed through post-market monitoring, periodic validation, and controlled model updates to ensure that system performance remains within predefined clinical limits.

To mitigate technical and clinical risks, several design and operational safeguards are implemented. Wearable subsystems incorporate guided sensor placement through dedicated textile structures, reducing the likelihood of incorrect positioning. The compression actuator includes multiple safety mechanisms, such as normally open valves for automatic pressure release in the event of power loss, a maximum pressure limit of 150 mmHg, and firmware-level protections including temporal constraints and redundant pressure sensing to prevent unsafe operation. The system operates under continuous human oversight, thereby limiting the clinical impact of both false positive detections (e.g., unnecessary therapeutic escalation) and false negatives (e.g., missed DVT), as all diagnostic interpretation and treatment decisions remain the responsibility of qualified healthcare professionals. Additional AI-specific safety and security risks are systematically identified and mitigated to ensure the reliability and trustworthiness of the system.

### Challenges and lessons learned

The development of the ThrombUS+ system revealed critical insights pertaining mainly to socio-technical alignment, technical component engineering, and the complexities of multipartner system integration. During the early design phases, aligning clinical expectations with technical possibilities emerged as an inherent challenge. This was addressed through a participatory, multidisciplinary approach that prioritized establishing a common language between medical and technical partners—a critical success factor for cohesive development. By involving practitioners from the outset, reference use cases were grounded in actual clinical practices rather than theoretical literature. This early collaboration enabled the team to identify usability and acceptability requirements with higher precision. To manage the resulting complexity of integrating diverse technologies, a structured schematic approach was employed to provide a clear overview of the multisensor system.

At the component level, several engineering obstacles highlighted the difficulty of continuous vascular monitoring. A primary hurdle involved the high-fidelity modeling of pathological conditions; existing tissue-mimicking phantoms often failed to capture the complex hemodynamic and structural characteristics of venous thrombosis required for laboratory testing. Furthermore, maintaining sensor interface stability over extended periods proved difficult, as stable acoustic coupling, consistent optical sensor pressure, and low bioimpedance were frequently compromised by variations in skin properties and patient motion. From a computational perspective, integrating real-time signal processing within resource-constrained wearable platforms required rigorous algorithmic optimization to balance analytical accuracy with energy efficiency. These issues were compounded by the clinical challenge of quantitatively estimating in vivo tissue response to compression, though preliminary tests confirmed that tissue compression from the back of the thigh is technically feasible via a simple pneumatic mechanism.

The final transition toward system integration and implementation provided essential lessons in collaborative manufacturing. A major technical challenge involved ensuring standardized hardware communication between modules developed by separate partners and the central software. A critical success factor in minimizing integration conflicts was the development of a functional software development kit template. This approach superseded static documentation as the primary tool for partner alignment, making the synchronization of diverse hardware modules considerably more efficient. Throughout the project, sharing nonfinal prototypes and utilizing 3D printing for rapid case development facilitated continuous iteration and improved mutual understanding across teams. However, while 3D printing was ideal for prototyping, its time-intensive nature for mass production led to gradual manufacturing and dispatch.

Despite these challenges, preliminary tests have confirmed that operator-less compression ultrasonography is feasible and can provide repeatable measurements under controlled conditions. These findings reinforce the potential of wearable ultrasound for continuous point-of-care thrombosis monitoring while highlighting the need for further work on dataset creation, model validation, and long-term usability.

### Expected outcomes and impacts

DVT and its complications, such as PE, can have substantial clinical and economic consequences. The cost and burden of DVT can be measured in several ways: direct healthcare costs, indirect costs due to loss of productivity, and the human toll in terms of morbidity and mortality. Direct healthcare costs involve the diagnosis, treatment, and management of DVT and its complications. Expenses can include hospitalizations, outpatient visits, anticoagulant medications, and follow-up care. DVT can lead to temporary or long-term disability, which can result in lost productivity, missed work, and additional costs for patients and their families. These indirect costs can be substantial, especially for individuals who develop post-thrombotic syndrome or chronic thromboembolic pulmonary hypertension. DVT can cause suffering and long-term health problems for affected individuals. Complications such as post-thrombotic syndrome can lead to chronic pain, swelling, and ulcers. The human burden of DVT and its complications cannot be directly translated into a monetary value, as it involves morbidity, mortality, and the emotional and psychological toll on patients and their families. However, it is important to recognize that the human burden is substantial, with DVT and PE affecting hundreds of thousands of individuals each year.

The European Union experiences a substantial DVT burden: an estimated 684,019 DVTs, 434,723 PEs, and 610,138 post-thrombotic syndrome events occur annually [47]. In a 2016 European study, DVT and its complications were estimated at €1.5 to €2.2 billion total annual costs, €1.0 to €1.5 billion of hospital-associated costs, €0.5 to €1.1 billion preventable costs, and €0.2 to €0.3 billion indirect costs, while indirect costs represented 12% of expenditures [48].

CDC estimated an annual average of 547,596 adult hospitalizations with venous thromboembolism in US, including 348,558 with DVT, 277,549 with PE, and 78,511 with both PE and DVT [49]. Annual DVT burden will continue to rise to 1.82 million events by the year 2050 in the United States [50]. Recent estimates suggest that the annual US costs of DVT ranged from $13.5 to $69.3 billion in 2011, of which $4.5 to $39.3 billion may have been preventable with improved prophylaxis measures [51]. The ThrombUS+ proposes a disruptive medical device technology, bringing high-quality diagnostic services to the nonexpert and the patient at the point of care. The system is engineered for versatility across various clinical workflows, including intraoperative monitoring during long surgeries, postoperative care in intensive care units, and continuous screening for bedridden patients in diverse settings such as oncology or perinatal wards. Beyond the hospital, the low-energy architecture promotes portability and eco-friendly operation, enabling use in remote environments, ambulances, or even during air travel.

In addition to its primary diagnostic and preventive goals, the ThrombUS+ aims to serve as a valuable research tool by generating large amounts of real-time health data. This information aids in the broader medical understanding of clot formation processes and patient rehabilitation. Furthermore, the project commits to open science by planning the large-scale collection and anonymization of data from over 3,000 patients, providing a valuable resource for the global research community to improve DVT outcomes and diagnostic accuracy.

Continuous monitoring enables early detection of thrombosis, facilitating prompt treatment and reducing the risk of adverse complications. Timely thrombosis treatment will not only reduce the risk of fatal complications such as PE, but it will also reduce the likelihood of long-term complications, such as developing post-thrombotic syndrome [52]. Consequently, continuous monitoring and early detection will have a direct, positive impact on patients’ long-term quality of life. In addition, the prevention and timely detection of DVT enabled by our device can facilitate early patient discharge, avoid unnecessary patient referrals, reduce patient travel time between hospital departments, and reduce the workload of healthcare personnel. Collectively, these benefits will have a positive social impact for both patients and healthcare providers, while also producing economic benefits for health systems by reducing hospitalization and rehabilitation costs. Finally, our device will allow for healthcare services in underserved areas.

Devices for point-of-care diagnosis are often intended for limited use, if not single use. They are made of potentially unsustainable materials and may require specialized disposal to avoid infection risk, thereby posing environmental concerns [53]. The ThrombUS+ device has been designed to allow sensors to be detached from the wearable components. This makes it feasible to use individual sensors separately, accounting for different patient needs. In addition, it allows the washing and disinfection of wearable parts, enabling multiple uses of the device, and underperforming or malfunctioning sensors can be replaced or updated individually if required, and the devices can be recycled at the end of their life cycle. Moreover, the smart textiles used are made from recycled, long-lasting, and hazardous-free materials.

The results of the ThrombUS+ project have the potential to deliver substantial socioeconomic benefits. Early detection and continuous monitoring of DVT can markedly reduce the incidence of severe complications such as PE, thereby lowering hospitalization rates and overall healthcare costs. The proposed technology facilitates a transition from reactive to preventive care, enhancing patient outcomes and quality of life while alleviating the burden on healthcare systems. Moreover, the adoption of such wearable monitoring devices can improve healthcare accessibility, particularly for patients in remote or resource-limited settings, by enabling decentralized and home-based diagnostics. On a broader scale, integrating multidisciplinary expertise in biomedical electronics, signal processing, and clinical research strengthens regional innovation capacity and stimulates economic growth by creating high-value jobs and new market opportunities in the medical technology sector.

## Discussion

Ultrasound imaging with simultaneous compression of veins is the primary method for diagnosing DVT [12]. However, it is time-consuming and requires highly skilled physicians. Image quality is crucial for accurate diagnosis yet remains a persistent challenge. To address this, an AI-based guidance system, ThinkSono, was recently introduced [54] to assist operators in acquiring higher-quality images [17]. Deep learning techniques have also been employed to classify ultrasound images based on their diagnostic adequacy [55]. Nevertheless, such tools remain assistive and rely on operator interaction.

In contrast, the ThrombUS+ solution aims to develop an operator-free ultrasound device for continuous monitoring of DVT risk. AI and machine learning are employed here not to assist operators but to enable the fully automated detection and segmentation of vein and thrombus images. This approach supports minimally obtrusive, long-term monitoring of potential DVT formation.

The ThrombUS+ enables continuous DVT monitoring at the most probable site of thrombus formation within the vein, which is particularly valuable in critical care settings, for example, in patients recovering from orthopedic surgery [56]. However, this method is limited in that it does not fully support operator-free localization of thrombi throughout the deep veins of the leg. To overcome this, the ThrombUS+ system incorporates additional subsystems based on impedance plethysmography and light-reflection rheography, enabling operator-free detection of DVT regardless of its location in the leg.

These subsystems go beyond current point-of-care DVT testing, which has recently attracted increasing attention [57] and also surpass current methods for screening asymptomatic patients [58]. The modular ThrombUS+ system comprises components for ultrasonic, impedance plethysmography, and LRR, making it adaptable across various clinical contexts. Its unobtrusive monitoring is achieved through a dedicated cuff equipped with inflatable bladders and controllable pressure, capable of compressing the vein to a degree that can temporarily occlude it. Silent, portable pumps and pressure sensors facilitate operator-free operation across all modalities. Simultaneous presentation of pressure values alongside ultrasound images enables estimation of vein and/or thrombus elasticity, offering a unique alternative to conventional elastography, which typically requires advanced-generation scanners [59]. Moreover, the ThrombUS+ functions as a key subsystem of a serious exergaming framework for DVT prevention, extending its role beyond monitoring to prophylaxis and rehabilitation.

At a broader level, ThrombUS+ should be positioned within the ongoing transformation of healthcare toward digital health ecosystems and hospital-at-home models. Unlike conventional diagnostic tools that operate at discrete time points, ThrombUS+ enables continuous, multimodal, and longitudinal monitoring, supporting a transition from reactive diagnostics to proactive and preventive care. This paradigm shift aligns with the emerging vision of decentralized healthcare, where high-risk patients can be safely monitored outside traditional hospital settings [60,61]. Within this context, several clinically relevant deployment scenarios emerge. In outpatient anticoagulation management, continuous monitoring could provide insight into thrombus progression or resolution, enabling more personalized and adaptive treatment strategies. In oncology ambulatory care, where patients face elevated thrombotic risk, the system could facilitate early detection of otherwise asymptomatic DVT events during treatment cycles. Similarly, in remote postoperative monitoring, particularly following early discharge after major surgery, ThrombUS+ could support continuous surveillance, reducing readmissions and enabling timely clinical intervention.

From a system-level perspective, the impact of ThrombUS+ extends beyond clinical performance to healthcare efficiency and economic outcomes. DVT and its complications impose a substantial burden on healthcare systems, with annual costs in Europe estimated at €1.5 to 2.2 billion [48]. By enabling continuous monitoring and earlier detection, ThrombUS+ may reduce severe complications such as PE, decrease hospitalization rates, and minimize long-term morbidity, while also reducing the need for repeated imaging, supporting earlier discharge, and improving resource allocation. Although formal health economic evaluation is beyond the scope of this study, these factors suggest a favorable cost-effectiveness profile, particularly in high-risk populations. A more structured assessment of the cost-effectiveness of the device will be performed in the later stages of the project, to better understand both the health and economic outcomes of the ThrombUS+ deployment. The data gathered from clinical study C2 will be used to estimate lifetime incremental quality adjusted life years (QUALY) and compare it with a standard intervention (non-ThrombUS+ intervention). Production and deployment costs based on early prototypes, together with QUALYs data, will then be analyzed through the Monitoring and Assessment Framework for the European Innovation Partnership on Active and Healthy Ageing tool to model the economic impact that the ThrombUS+ device can have on healthcare systems.

The advanced operator-independent monitoring mode and the unobtrusive operation of the ThrombUS+ system inevitably introduce certain limitations. One such limitation is the reduced ability to localize thrombi within the veins precisely. While the ultrasound transducer is positioned to detect thrombi with relatively high probability, a thrombus may still form elsewhere. The ThrombUS+ can detect thrombus anywhere in the leg using impedance plethysmography and LRR; however, determining its exact location remains a challenge.

## Conclusion

The ThrombUS+ system analysis concluded that effective DVT monitoring requires a universal, multimodal approach that integrates compression ultrasonography, electrical impedance plethysmography, and LRR within a shared architecture. This architecture comprises a wearable actuator module, a central hub with specialist software, and an XR/SG module for patient prophylaxis. The project introduces several innovations, the most notable being operator-independent, AI-driven CUS for automated DVT detection; however, its technical feasibility depends on vein visibility during compression dynamics. Operator independence will also be pursued in other modalities, including electrical impedance plethysmography and LRR. Critical knowledge gaps remain regarding optimal patient positioning, sensor placement protocols, and venous flow thresholds for reducing thromboembolism risk during continuous monitoring. These gaps will be addressed through carefully planned preclinical and clinical studies.
